# Länger gesund im Beruf? Trends in den Lebensjahren frei von Herz-Kreislauf- und Muskel-Skelett-Erkrankungen in GKV-Daten für die Erwerbstätigen- und Allgemeinbevölkerung

**DOI:** 10.1007/s00103-024-03868-8

**Published:** 2024-04-12

**Authors:** Juliane Tetzlaff, Jelena Epping

**Affiliations:** Medizinische Soziologie, Medizinische Hochschule Hannover, Carl-Neuberg-Str. 1, 30625 Hannover, Niedersachsen Deutschland

**Keywords:** Muskel-Skelett-Erkrankungen, Herz-Kreislauf-Erkrankungen, Erkrankungsfreie Lebenserwartung, Berufliche Ungleichheiten, Krankenkassendaten, Musculoskeletal diseases, Cardiovascular diseases, Disease-free life expectancy, Occupational inequalities, Health insurance claims data

## Abstract

**Hintergrund:**

Unsere Studie fokussiert auf die Entwicklung der erkrankungsfreien Lebensjahre in Bezug auf 2 Erkrankungsgruppen mit hoher Public-Health-Relevanz: Muskel-Skelett- (MSE) und Herz-Kreislauf-Erkrankungen (HKE). Vor dem Hintergrund der steigenden Lebensarbeitszeit wird die Entwicklung der erkrankungsfreien Jahre der Bevölkerung im erwerbsfähigen Alter und der Erwerbstätigen verglichen und Unterschiede zwischen den Berufsgruppen beleuchtet.

**Methoden:**

Die Studie basiert auf den Daten der AOK Niedersachsen (*N* = 2.001.225). Die Erkrankungsfälle werden anhand der Diagnosedaten ermittelt. Die erwarteten Jahre frei von MSE und HKE werden mit Multistate-Life-Table-Analysen für 3 Perioden zwischen 2006 und 2018 berechnet. Die Berufsgruppe wird über den Berufsschlüssel definiert und in 3 Gruppen unterteilt: 1) Un- und Angelernte, 2) Fachkräfte und Spezialisten, 3) Hochqualifizierte.

**Ergebnisse:**

Die Lebensjahre frei von MSE nahmen in der Allgemeinbevölkerung und unter den Erwerbstätigen deutlich ab. Die stärkste Verringerung zeigte sich in der höherqualifizierten Berufsgruppe. Die Lebensjahre frei von HKE stiegen in der Allgemeinbevölkerung im Zeitverlauf. Die Zunahmen waren unter den Erwerbstätigen geringer, sie wurden nur bei Männern in un- und angelernter Tätigkeit verzeichnet.

**Diskussion:**

Die Studie zeigt, dass sich die erkrankungsfreien Jahre unter den Erwerbstätigen teilweise schlechter als in der Allgemeinbevölkerung entwickelten. Für die untersuchten Erkrankungsgruppen MSE und HKE bestehen deutliche Ungleichheiten zwischen den Berufsgruppen, die sich im Zeitverlauf etwas verringerten. Die Ungleichheiten und die Abnahme der Jahre frei von MSE belegen die hohe Public-Health-Relevanz und den Bedarf an Präventionsmaßnahmen zur Vermeidung von HKE und MSE im erwerbsfähigen Alter.

**Zusatzmaterial online:**

Zusätzliche Informationen sind in der Online-Version dieses Artikels (10.1007/s00103-024-03868-8) enthalten.

## Abstract (en)

**Background:**

Our study focuses on the development of disease-free life years of two disease groups with high public health relevance: musculoskeletal diseases (MSD) and cardiovascular diseases (CVD). Against the backdrop of prolonged working lives, the development of disease-free life years in the working-age population is compared with the trend in the employed population. Differences between occupational groups are also examined.

**Methods:**

The study is based on data from the statutory health insurance provider AOK Lower Saxony (*N* = 2,001,225). Incident cases were identified based on the diagnosis data. The expected years free of MSD and CVD were calculated using multistate life table analysis for three periods between 2006 and 2018. The occupational group is identified via the occupational key. Three groups are distinguished: unskilled and semi-skilled workers, skilled workers and specialists, and highly skilled workers.

**Results:**

Life years free of MSD clearly decreased in the general population and among the employed population. The decrease was strongest in the higher-skilled occupational groups. Life years free of CVD increased in the general population. The increase was weaker among the employed population. The only occupational group showing increases were men in unskilled and semi-skilled occupations.

**Discussion:**

The study shows that disease-free life years among employed persons developed in some cases worse than in the general population. For the analysed disease groups MSD and CVD, there are clear inequalities between occupational groups, which decreased somewhat over time. The inequalities and the decrease in years free of MSD highlight the high public health relevance and the need for effective prevention strategies to prevent CVD and MSD in working age.

## Einleitung

Wie in vielen anderen westlichen Ländern wird auch in Deutschland die Bevölkerung insgesamt älter. Vor dem Hintergrund der steigenden Lebenserwartung stellt sich die Frage, ob die gewonnenen Lebensjahre in Gesundheit oder in Morbidität verbracht werden. Diese Studie fokussiert auf Herz-Kreislauf-Erkrankungen und auf Erkrankungen des Muskel-Skelett-Systems als 2 Erkrankungsgruppen, die aufgrund ihrer weiten Verbreitung in der Bevölkerung [1, 2] und ihrer Folgen für die betroffenen Personen [3–5] eine hohe Public-Health-Relevanz besitzen. Wir analysieren, wie sich die erkrankungsfreien Lebensjahre im Kontext der im Zeitverlauf steigenden Lebenserwartung entwickelt haben. Da beide Erkrankungsgruppen auch bereits im mittleren Erwachsenenalter auftreten, liegt ein besonderes Augenmerk der Studie auf dem Vergleich der Entwicklung in der Allgemeinbevölkerung und unter den Erwerbstätigen. Die Erwerbstätigen können aufgrund des steigenden Renteneintrittsalters und der sich verändernden Beschäftigungsbedingungen [6] als eine Gruppe angesehen werden, die besonderen Belastungen ausgesetzt ist, was sich negativ auf die Gesundheit auswirken kann.

Zur Frage, wie sich die erkrankungsfreie Lebenszeit bei steigender Lebenserwartung entwickelt, existieren 2 konträre Thesen: Die Expansionsthese [7] nimmt an, dass durch eine verbesserte medizinische Versorgung die Überlebenszeit nach dem Erkrankungseintritt zunimmt, wodurch sich die gesunde Lebenszeit im Zeitverlauf absolut oder anteilig verkürzt. Die Kompressionsthese [8] sieht eine wirksame Prävention als treibenden Faktor für den Anstieg der gesunden Lebensjahre an, wodurch eine Komprimierung der morbiden Lebensjahre zum Ende des Lebens hin ermöglicht wird. Während bereits zahlreiche Studien die Entwicklung der Lebenszeit frei von Behinderungen und funktionalen Einschränkungen [9–11] oder einer schlechten subjektiven Gesundheit [12, 13] untersuchten, bleibt weiterhin unklar, welche Erkrankungen sich dahinter verbergen. Studien zur Entwicklung der Lebenszeit frei von spezifischen Erkrankungen sind bisher selten [14–17].

Muskel-Skelett-Erkrankungen (MSE) stellen weltweit eine der Hauptursachen von chronischem Schmerz und Behinderungen dar [3, 18]. Sie belasten die Betroffenen auch im Erwerbsalter oft so stark, dass ein vorzeitiges Ausscheiden aus dem Arbeitsmarkt wahrscheinlicher wird [4, 19]. In Deutschland sind sie eine der häufigsten Ursachen für Arbeitsunfähigkeit [20]. Herz-Kreislauf-Erkrankungen (HKE) stellen ebenfalls häufig eine starke Belastung für die Betroffenen dar, erhöhen das Risiko für Herzinfarkt und Schlaganfall und sind ebenfalls mit einer vorzeitigen Aufgabe der Erwerbstätigkeit assoziiert [21]. Beide Erkrankungsgruppen besitzen daher eine besondere Public-Health-Relevanz, die sich nicht nur auf die Allgemeinbevölkerung, sondern auch auf die Erwerbstätigenbevölkerung erstreckt. Aufgrund der verlängerten Lebensarbeitszeit [10, 22–24], der Zunahme von atypischer und prekärer Beschäftigung und der Verdichtung von Arbeit in vielen Bereichen sind Erwerbstätige zahlreichen Belastungen ausgesetzt, die sich negativ auf ihre Gesundheitstrends auswirken können [6, 25, 26]. Da sich die beruflichen Belastungen zwischen den unterschiedlichen Berufsgruppen unterscheiden, fokussiert unser Paper auch auf die Unterschiede in der Entwicklung der erkrankungsfreien Lebensjahre zwischen den Berufsgruppen.

Die Analyse der Lebensjahre frei von spezifischen Erkrankungen trägt in mehrfacher Hinsicht zur Erweiterung des bisherigen Forschungsstandes bei. Wenn allgemeine Gesundheitsindikatoren wie selbstberichtete Gesundheit oder funktionale Einschränkungen betrachtet werden, bleiben die dahinterliegenden spezifischen Erkrankungen unbekannt. Die Analyse spezifischer Erkrankungen ermöglicht es, erkrankungsspezifische Präventionsbedarfe aufzudecken. Die Studie nimmt außerdem die Gruppe der Erwerbstätigen in den Blick, die als wachsende und alternde Gruppe in der Bevölkerung eine steigende Public-Health-Relevanz aufweist. Die Studie greift folgende Forschungsfragen auf:

- Wie entwickelt sich die Lebenszeit frei von Muskel-Skelett-Erkrankungen und Herz-Kreislauf-Erkrankungen in der Allgemein- und Erwerbstätigenbevölkerung? Unterscheiden sich die Entwicklungen?
- Bestehen Unterschiede in den erkrankungsfreien Lebensjahren zwischen den Berufsgruppen? Wie entwickeln sich diese Ungleichheiten über die Zeit?

## Methoden

### Daten

Die Analysen beruhen auf den Abrechnungsdaten der Gesetzlichen Krankenversicherung (GKV) AOK Niedersachsen der Jahre 2005–2018 für alle versicherten Personen zwischen 30 und 65 Jahren (*N* = 2.001.225). Der Datensatz beinhaltet neben den Versicherungsverläufen auch Angaben zur Mortalität, zum Erwerbsstatus (z. B. erwerbstätig, Arbeitslosengeld I/II, berentet, familienversichert) sowie detaillierte Informationen zu ambulanten und stationären Diagnosen (kodiert nach der 10. Internationalen Klassifikation der Krankheiten, deutsche Version – ICD-10 GM). Aufgrund dieser vielfältigen Informationen zur Morbidität und Mortalität in Verbindung mit den Individualinformationen zur Erwerbstätigkeit und zum sozioökonomischen Status ist diese Datenbasis besonders gut für die hier durchgeführten Zeittrendanalysen geeignet.

Für erwerbstätige Versicherte ist außerdem der aktuell ausgeübte Beruf nach der Klassifikation der Berufe 1992 und 2010 (KldB-92, KldB-2010) enthalten. Dieser wird in Deutschland regelmäßig und gesetzlich verpflichtend durch den Arbeitgeber an die Krankenkasse gemeldet. Auf der Basis dieser Berufsschlüssel wurde für die Analysen die Kategorisierung der Berufe in 3 Gruppen vorgenommen: 1) Un- und Angelernte (einfache Tätigkeiten), 2) Fachkräfte und Spezialisten und 3) Hochqualifizierte [27]. Innerhalb einer Berufsgruppe ähneln sich die Berufe hinsichtlich ihres üblicherweise zur Ausübung des Berufs benötigten Schulabschlusses, des Anforderungsniveaus und der Art der Tätigkeit.

Die Versichertenpopulation der AOK Niedersachsen ist hinsichtlich der Alters- und Geschlechterstruktur vergleichbar mit der gesamtdeutschen und der niedersächsischen Bevölkerung [28, 29]. Wie in jeder gesetzlichen Krankenkasse zu erwarten, sind Erwerbstätige in hochqualifizierten Berufen unterrepräsentiert [29]. Diesem Umstand wird durch die Stratifizierung in den Analysen Rechnung getragen. Um die Entwicklung der erkrankungsfreien Lebenszeit in der Allgemein- und Erwerbstätigenbevölkerung im Zeitverlauf abzubilden, wurden die Daten in 3 Perioden eingeteilt: 2006–2008, 2011–2013 und 2016–2018. In der Allgemeinbevölkerung im Alter 30 bis 65 Jahre stellen die sozialversicherungspflichtig Beschäftigten die größte Gruppe. Je nach betrachteter Altersgruppe und Periode schwankt ihr Anteil in den Daten der AOK Niedersachsen zwischen 31 % (Frauen, 55–64 Jahre, 2008) und 78 % (Männer, 35–44 Jahre, 2018). Ähnliche, wenn auch etwas geringere Anteile finden sich auch in der deutschen Bevölkerung unter den Männern. Bei den Frauen sind die Anteile unter den AOK-Versicherten etwas geringer als in der deutschen Bevölkerung (s. Tabelle S1 im Onlinematerial zu diesem Beitrag).

### Inzidenz von Muskel-Skelett- und Herz-Kreislauf-Erkrankungen

Zur Operationalisierung der Inzidenzraten von Muskel-Skelett- und Herz-Kreislauf-Erkrankungen wurden stationäre und ambulante Diagnosecodes verwendet. Eine Person galt dann als inzidenter Fall, wenn eine Diagnose in mindestens 2 Quartalen eines Jahres kodiert wurde. Eine Ausnahme hiervon bilden Herzinfarkt und Schlaganfall, für die – wie auch in anderen Studien üblich – eine einmalige Nennung als stationäre Hauptdiagnose ausreichte, um einen Fall zu definieren. Fälle von neuauftretenden Akutereignissen, bei denen die betreffende Person vor der stationären Aufnahme verstorben ist, können in den Daten in der Regel nicht identifiziert werden. Eine vollständige Liste der Diagnosecodes der Muskel-Skelett- und Herz-Kreislauf-Erkrankungen ist in Tab. 1 einsehbar. Die Auswahl der HKE-Diagnosen erfolgte nach Konsultation von klinischen Expert:innen mit Fokus auf potenzielle Einschränkungen der Erwerbsfähigkeit. Dieses Vorgehen der Validierung der Diagnosen ist etabliert und entspricht der Guten Praxis der Sekundärdatenanalyse [30].

**Table 1:** 

*Muskel-Skelett-Erkrankungen*	
Arthropathien	M00-25
Systemkrankheiten des Bindegewebes	M30-36
Krankheiten der Wirbelsäule und des Rückens	M40-54
Krankheiten der Weichteilgewebe	M60-79
Osteopathien und Chondropathien	M80-94
Sonstige Krankheiten des Muskel-Skelett-Systems und des Bindegewebes	M95-99
*Herz-Kreislauf-Erkrankungen*	
Koronare Herzkrankheiten (inkl. Herzinfarkt)	I20-25
Herzinsuffizienz	I50
Schlaganfall	I60-64
Periphere arterielle Verschlusskrankheit	I70.2 und I73.9

Um zwischen prävalenten und inzidenten Fällen zu differenzieren, hat sich die Anwendung von einjährigen Vorbeobachtungszeiten etabliert [31]. Entsprechend gilt eine Person nur dann als inzident, wenn der individuellen Erstdiagnose 365 diagnosefreie Versicherungstage vorausgingen. Da einige der am häufigsten auftretenden Muskel-Skelett-Erkrankungen nicht chronisch progressiv verlaufen, sondern eine vollständige Genesung erlauben (z. B. Rücken- oder Muskelschmerz), wurde für diese Erkrankungsgruppe auch die Genesung berücksichtigt. Hierfür mussten Personen ein Jahr lang frei von weiteren Diagnosen aus dem Bereich der Muskel-Skelett-Erkrankungen (M00-99) sein. Da die eingeschlossenen Herz-Kreislauf-Erkrankungen entweder chronisch progredienter Natur sind oder Akutereignisse darstellen, die sehr häufig mit schwerwiegenden und langanhaltenden Folgen für die Betroffenen verbunden sind (z. B. Herzinfarkt, Schlaganfall), wird die Genesung bei Herz-Kreislauf-Erkrankungen aus inhaltlichen Überlegungen heraus nicht definiert.

### Statistische Methoden

Die Berechnung der erkrankungsfreien Jahre erfolgte mittels Multistate-Life-Table-Analysen, die analog zur von Palloni vorgeschlagenen Methode des Increment-Decrement Life Table [32] auf beobachteten (und nicht auf geschätzten) altersspezifischen Übergangsraten zwischen unterschiedlichen Status basieren. Mit dieser Methode können die erkrankungsfreien Lebensjahre als Teilmenge der gesamten Lebenserwartung berechnet werden. In Anlehnung an das Konzept der Lebenserwartung erhält man so die Lebensjahre frei von Erkrankung, die unter den zum betrachteten Zeitpunkt geltenden Übergangsraten zu erwarten sind. Diese Übergangsraten wurden neben der Gesamtgruppe der 30–65-Jährigen auch für die Erwerbstätigen sowie für die einzelne Berufsgruppen berechnet.

Bei Herz-Kreislauf-Erkrankungen wurden 3 Übergänge einbezogen: 1) die Inzidenz (Übergang vom Status „erkrankungsfrei“ zu „erkrankt“), 2) das Versterben mit der Erkrankung (prävalent zu Tod) und 3) das Versterben ohne die Erkrankung (nicht prävalent zu Tod). Bei den Muskel-Skelett-Erkrankungen wurde zusätzlich zu den 3 genannten Übergängen die Genesung als weiterer Übergang berücksichtigt (prävalent zu nicht prävalent).

Die entsprechenden Übergangsraten wurden altersspezifisch berechnet, indem die beobachtete Anzahl des jeweiligen Ereignisses (z. B. Inzidenz) in der jeweiligen Altersgruppe ins Verhältnis zur beobachteten Risikozeit (ereignisfreie Zeit) gesetzt wurde. Anschließend wurden die Übergangsraten jeder Altersgruppe dafür verwendet, für jede Subgruppe die Anzahl der Jahre frei von MSE oder HKE im Alter 30 zu berechnen, die bis zum 65. Lebensjahr statistisch zu erwarten sind. Darüber hinaus wurde auch die Health Ratio (Anteil der weiteren Lebensjahre frei von Erkrankung an den insgesamt bis zum Alter 65 zu erwartenden Lebensjahren) für die Allgemeinbevölkerung berechnet.

Die Berechnungen erfolgten auf Basis von 5‑Jahres-Altersstufen, um auch für seltenere Übergänge (z. B. Versterben im jüngeren Alter) ausreichend Fälle für die Berechnung der altersspezifischen Übergangsraten zur Verfügung zu haben. Um die Ergebnisse der Allgemeinbevölkerung mit jenen der Erwerbstätigen vergleichen zu können, wurden in beiden Gruppen Personen bis zum 65. Lebensjahr betrachtet. Daher sind alle Lebensjahre als partielle (oder temporäre) Lebenserwartung bis zum Alter 65 zu interpretieren. Aufgrund der sehr geringen Inzidenz von Herz-Kreislauf-Erkrankungen unterhalb des 30. Lebensjahres und der verlängerten Ausbildungszeiten für Hochqualifizierte wurde die untere Altersuntergrenze auf 30 Jahre gelegt.

Die Berechnungen wurden mit Stata MP 16.0 und R 3.5.1 durchgeführt. Die 95 %-Konfidenzintervalle basieren auf 1000 Bootstraps. Hierfür wurden für jede der betrachteten Subgruppen 1000 Samples aus dem Gesamtdatensatz gezogen und die erkrankungsfreien Jahre für jedes Sample berechnet. Aus der resultierenden Verteilung der erkrankungsfreien Jahre wurden die 2,5 % mit den höchsten bzw. den niedrigsten Werten ausgeschlossen, die übrige Spannbreite bildet das 95 %-Konfidenzintervall.

## Ergebnisse

In der Tab. 2 sind die den Analysen zugrunde liegenden Fallzahlen dargestellt. Aufgrund des Ausschlusses von prävalenten Fällen in der jeweiligen Erkrankungsgruppe (s. Daten und Methoden) unterscheiden sich die MSE- und HKE-Populationen voneinander. Tabelle S2 (s. Onlinematerial) zeigt außerdem, dass alle betrachteten Übergangsraten den erwarteten Anstieg über das Alter hinweg aufweisen.

**Table 2:** 

		2006–2008		2011–2013		2016–2018	
		Weiblich	Männlich	Weiblich	Männlich	Weiblich	Männlich
Muskel-Skelett-Erkrankungen							
Allgemeinbevölkerung	Personenjahre	934.003	1.056.624	977.531	1.157.013	1.056.182	1.261.372
	Rohe Inzidenz pro 100.000	16.461	13.919	16.589	14.346	16.654	14.152
Erwerbstätige: un- und angelernte Berufe	Personenjahre	194.246	311.462	171.567	200.820	189.424	219.450
	Rohe Inzidenz pro 100.000	18.847	15.528	19.644	16.117	19.240	15.235
Erwerbstätige: Fachkräfte und Spezialisten	Personenjahre	210.170	340.248	306.753	560.088	401.680	623.403
	Rohe Inzidenz pro 100.000	17.006	13.961	17.015	15.071	17.062	15.056
Erwerbstätige: Hochqualifizierte	Personenjahre	13.006	15.421	26.310	29.230	38.288	41.543
	Rohe Inzidenz pro 100.000	13.563	9533	13.983	9976	13.673	10.327
Herz-Kreislauf-Erkrankungen							
Allgemeinbevölkerung	Personenjahre	1.399.029	1.368.921	1.570.431	1.584.177	1.742.209	1.766.903
	Rohe Inzidenz pro 100.000	982	1625	893	1545	928	1501
Erwerbstätige: un- und angelernte Berufe	Personenjahre	262.980	361.172	253.670	241.517	292.842	274.756
	Rohe Inzidenz pro 100.000	762	1318	793	1234	888	1171
Erwerbstätige: Fachkräfte und Spezialisten	Personenjahre	291.951	420.904	449.444	734.999	612.797	846.705
	Rohe Inzidenz pro 100.000	478	923	494	1105	540	1165
Erwerbstätige: Hochqualifizierte	Personenjahre	16.669	17.170	35.786	33.962	52.185	49.146
	Rohe Inzidenz pro 100.000	318	646	363	627	372	739

**Figure 1:**
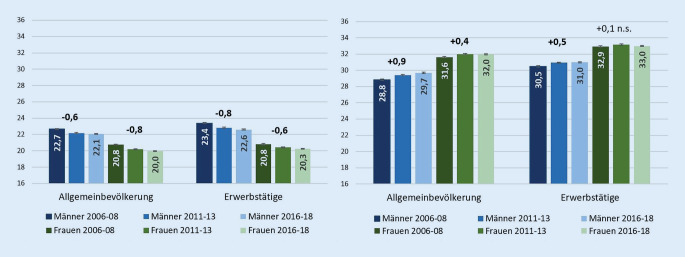

Im ersten Schritt wurden die Entwicklungen der Lebensjahre frei von Muskel-Skelett- und Herz-Kreislauf-Erkrankungen in der Allgemein- und Erwerbstätigenbevölkerung miteinander verglichen. 2006–2008 konnten Männer und Frauen in der Allgemeinbevölkerung im Alter von 30 Jahren noch 22,7 bzw. 20,8 Jahre frei von Muskel-Skelett-Erkrankungen erwarten (Abb. 1). Diese Werte wurden mit den insgesamt bis zum Alter 65 zu erwartenden Lebensjahren in der Allgemeinbevölkerung ins Verhältnis gesetzt (Health Ratio). Bei Männern sank die Health Ratio für MSE zwischen den Perioden 2006–2008 und 2016–2018 von 64 % auf 62 %, bei Frauen von 58 % auf 56 %. Eine andere Entwicklung fand bei HKE statt: Hier stieg die Health Ratio über die Zeit von 81 % auf 83 % bei Männern und von 88 % auf 89 % bei Frauen.

In der Erwerbstätigenbevölkerung lag die Anzahl der zu erwartenden Jahre frei von MSE für Männer bei 23,4 und für Frauen unverändert bei 20,8 Jahren (Abb. 1). 10 Jahre später war die Lebenserwartung frei von MSE bei Männern in der Allgemeinbevölkerung um 0,6 und unter den Erwerbstätigen um 0,8 Jahre geringer. Bei Frauen konnte eine sehr ähnliche Entwicklung beobachtet werden. Die Veränderung der Lebensjahre frei von MSE über die Zeit ist zudem statistisch signifikant.

Für HKE sehen wir bei höherem Niveau der erkrankungsfreien Lebensjahre einen umgekehrten Trend, der bei Frauen und Männern in der Allgemeinbevölkerung als auch bei Männern in der Erwerbstätigenbevölkerung signifikante Zunahmen aufzeigt (Abb. 1). Der Anstieg in den Lebensjahren frei von HKE fällt unter den Erwerbstätigen etwas schwächer aus als in der Allgemeinbevölkerung.

**Figure 2:**
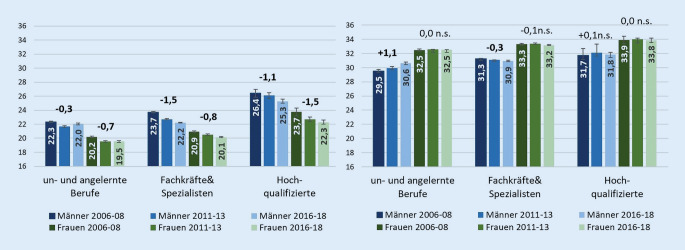

In den Lebensjahren frei von MSE und HKE für die Erwerbstätigenbevölkerung zeigt sich insgesamt ein klarer sozialer Gradient zwischen den Berufsgruppen. Personen in hochqualifizierten Berufen können bis zu 4,1 mehr erkrankungsfreie Jahre (MSE, Männer) erwarten als Personen anderer Berufsgruppen. Die Abnahme in den Lebensjahren frei von MSE fällt bei erwerbstätigen Frauen am stärksten in hochqualifizierten Berufen aus (23,7 auf 22,3 Jahre; Abb. 2). Bei Männern ist der Rückgang in den Lebensjahren frei von MSE am deutlichsten unter den Fachkräften und Spezialisten (23,7 auf 22,2 Jahre) ausgeprägt. Am wenigsten durch den negativen Gesundheitstrend in den Muskel-Skelett-Erkrankungen betroffen waren berufstätige Männer in un- und angelernten Berufen. Auch bei den Lebensjahren frei von HKE profitieren berufstätige Männer in un- und angelernten Berufen überdurchschnittlich stark. Bei allen anderen Gruppen ergaben sich keine nennenswerten Veränderungen. Bei beiden Erkrankungsgruppen verringerten sich die sozialen Ungleichheiten in den erkrankungsfreien Jahren unter den Erwerbstätigen. Eine Ausnahme hiervon bilden die HKE-freien Jahre bei den Frauen.

## Diskussion

Die Studie fokussiert auf 2 für die Erwerbsfähigkeit hochrelevante Erkrankungsgruppen, deren Inzidenz sich in der Bevölkerung über die Zeit konträr entwickelt hat. Während bei Herz-Kreislauf-Erkrankungen ein Rückgang der Morbidität und Mortalität berichtet wird [16, 17, 33–35], steigt die Zahl der AU-Tage und die Zahl der Diagnosen und Medikamentenverordnungen bei Muskel-Skelett-Erkrankungen an [1, 20, 36]. Diese Entwicklungen haben einen deutlichen Einfluss auf die erkrankungsfreien Lebensjahre in der Allgemein- und der Erwerbstätigenbevölkerung. Die Studie wurde vor dem Hintergrund der verlängerten Lebensarbeitszeit [10, 22, 24], der gestiegenen Erwerbsbeteiligung [37, 38], veränderterer Arbeitsmarktbedingungen und Tätigkeitsprofile [6, 25, 39] sowie der zunehmend restriktiveren Zugangsmöglichkeiten zur Frühberentung und Erwerbsminderungsrente [40, 41] durchgeführt. Dabei wurde untersucht, ob sich die erkrankungsfreien Lebensjahre von Erwerbstätigen im Zeitverlauf aufgrund dieser Veränderungen möglicherweise sogar schlechter entwickelt haben als in der Allgemeinbevölkerung.

Dies zeigt sich im Falle der Muskel-Skelett-Erkrankungen nicht: Die erkrankungsfreien Lebensjahre nehmen im Zeitverlauf bei Erwerbstätigen ab und spiegeln somit den negativen Gesundheitstrend in der Gesamtbevölkerung wider. Entsprechend kann davon ausgegangen werden, dass sich die oben genannten strukturellen Veränderungen auf dem Erwerbsmarkt bisher nicht stärker nachteilig auf die muskuloskelettale Gesundheit der Erwerbstätigen auswirkten als auf die muskuloskelettale Gesundheit der Allgemeinbevölkerung. Hierbei kann diskutiert werden, ob Veränderungen mit nachteiligem Effekt (wie z. B. der erschwerte Zugang zur Frühverrentung) durch Veränderungen mit positiven Effekten auf die Gesundheit der Erwerbstätigen (z. B. verbesserter Arbeitsschutz) ganz oder teilweise ausgeglichen wurden. Bei Herz-Kreislauf-Erkrankungen steigt die Anzahl der erkrankungsfreien Lebensjahre über die Zeit leicht an. Dieser Anstieg fällt unter den erwerbstätigen Männern jedoch etwas schwächer aus als in der Allgemeinbevölkerung. Bei erwerbstätigen Frauen konnte kein Anstieg der erkrankungsfreien Lebenszeit gefunden werden. Dies muss vor dem Hintergrund der bereits sehr hohen erkrankungsfreien Lebenszeit bei Frauen bis zum 65. Lebensjahr betrachtet werden, die nur begrenzt Raum für weitere Anstiege zulässt. Die Annahme, dass sich die gestiegene Erwerbsbeteiligung, insbesondere in höherem Alter und bei Frauen [10], negativ auf die kardiovaskuläre Gesundheit der Erwerbstätigen auswirken könnte, konnte nicht bestätigt werden.

Die erkrankungsfreien Lebensjahre unter den Erwerbstätigen entwickeln sich jedoch nicht in allen Berufsgruppen gleich. So bleiben die erwarteten Jahre frei von MSE unter den Männern mit einfachen Tätigkeiten nahezu konstant, während sie in der Gruppe der Spezialisten und Hochqualifizierten deutlich abnahmen. Unter den Frauen finden sich die stärksten Rückgänge in den Jahren frei von MSE ebenfalls in den beiden höherqualifizierten Berufsgruppen, wodurch sich die Ungleichheiten insgesamt verringern. Ein Grund für die weniger ausgeprägten Rückgänge in niedrigqualifizierten und industrienahen Berufen könnte in verbesserten Arbeitsbedingungen in diesem Sektor liegen, die sich durch die zunehmende Automatisierung, die Digitalisierung und den verbesserten Arbeitsschutz [39, 42] positiv auf die muskuloskelettale Gesundheit ausgewirkt haben könnten. Gleichzeitig könnten die zunehmende Dauer von sitzenden Tätigkeiten im Beruf und Alltag [43, 44] und die zunehmenden Adipositasraten [45] zum Absinken der erkrankungsfreien Lebenszeit in höherqualifizierten Berufen beigetragen haben. Der Beitrag dieser Einflussfaktoren wurde jedoch noch nicht ausreichend untersucht.

Neuere Studien zeigen, dass der stark verlangsamte Rückgang der Herz-Kreislauf-Sterblichkeit in den letzten Jahren maßgeblich zur Vergrößerung der „Lücke“ in der Lebenserwartung zwischen Deutschland und anderen europäischen Ländern und zur bestehenden sozialen Ungleichheit in der Lebenserwartung innerhalb Deutschlands beiträgt [46–48]. Dies hebt die zentrale Bedeutung der Prävention von kardiovaskulären Erkrankungen hervor. Die in der Studie eingeschlossenen Herz-Kreislauf-Erkrankungen treten unter den Erwerbstätigen nach wie vor recht selten auf, wodurch die erkrankungsfreie Lebenszeit in dieser Gruppe bereits hoch ist und im Zeitverlauf kaum noch anstieg. Dennoch bestehen auch hier deutliche Ungleichheiten zwischen den Berufsgruppen, was die Potenziale für den Zugewinn an weiteren Lebensjahren frei von Herz-Kreislauf-Erkrankungen insbesondere in der Gruppe von Männern in Berufen mit einfachen Tätigkeiten unterstreicht. Das erhöhte Risiko für kardiovaskuläre Erkrankungen wurde in der Medizin jedoch zumindest teilweise erkannt und ein stärkeres Augenmerk auf die Versorgung von Männern gelegt [49]. Dies dürfte auch zu einer Verbesserung der Versorgung von Männern mit niedrigem sozialen Status geführt und somit den Zugewinn der erkrankungsfreien Jahre unter den Männern in Berufen mit einfachen Tätigkeiten begünstigt haben.

### Stärken und Limitationen

Die vorliegende Studie basiert auf Routinedaten der gesetzlichen Krankenkasse AOK Niedersachsen des Zeitraums von 2005 bis 2018 und ist daher nicht von Erinnerungs‑, Response- oder Teilnahmebias beeinflusst [50]. Hierdurch konnten im Unterschied zu Befragungsstudien auch Diagnoseinformationen von Personen mit schweren Erkrankungen unverzerrt berücksichtigt werden [51]. Die Daten enthalten außerdem detaillierte Informationen über die Beschäftigungsdauer und -art der versicherten Personen über den gesamten Studienzeitraum, was für die durchgeführten Analysen unerlässlich ist. Frühere Studien [28, 29] überprüften die Repräsentativität der Daten hinsichtlich der Erwerbstätigenanteile sowie der Geschlechter- und Altersverteilung und zeigten hinsichtlich der demografischen Merkmale eine gute Vergleichbarkeit mit der deutschen und niedersächsischen Allgemeinbevölkerung. Um die bestehenden Unterschiede hinsichtlich der Sozialstruktur auszugleichen, wurden die Analysen stratifiziert nach Berufsgruppen durchgeführt. Die verwendeten Bildungsgruppen garantieren zudem eine ausreichend hohe Fallzahl innerhalb der jeweiligen Gruppe. Bezüglich der Analysen der Allgemeinbevölkerung muss aufgrund der Überrepräsentanz von Personen mit niedriger beruflicher Qualifikation in den Daten jedoch von einer erhöhten Morbidität ausgegangen werden. Die Anzahl der erwarteten erkrankungsfreien Lebensjahre ist hierdurch im Vergleich zur niedersächsischen bzw. deutschen Allgemeinbevölkerung vermutlich unterschätzt. So zeigte eine vorangegangene Studie eine geringere Lebenserwartung der Versicherten der AOK Niedersachsen als in der allgemeinen deutschen Bevölkerung [52]. Die vorliegende Studie fokussiert allerdings auf die Zeittrends in den erkrankungsfreien Lebensjahren, die bei einer gleichbleibenden Überrepräsentanz von Personen mit niedrigem sozioökonomischen Status robust dargestellt werden können. Hinweise auf eine gleichbleibende Berufsstruktur der erwerbstätigen Versichertenpopulation lassen sich in entsprechenden Repräsentativitätsanalysen finden [29].

Aufgrund der Breite der in dieser Studie berichteten Ergebnisse wurden umfassendere Berufsgruppen für die Analysen gewählt. Es kann dabei nicht ausgeschlossen werden, dass sich die beruflichen Belastungen innerhalb der breiteren Gruppen unterscheiden und sich die berufsbezogenen Auswirkungen auf die muskuloskelettale Gesundheit oder die Herz-Kreislauf-Gesundheit möglicherweise etwas nivellieren könnten. Für den Blick auf feinere Berufsgruppen müsste die Analyse aufgrund der geringen Anzahl der Ereignisse im jüngeren Alter jedoch auf höhere Altersstufen beschränkt werden.

Da es sich um Abrechnungsdaten handelt, kann nicht ausgeschlossen werden, dass Personen ohne aktuell behandlungsbedürftige Erkrankung als Fälle markiert wurden. Außerdem sollte berücksichtigt werden, dass sich die Kodierpraxis in Arztpraxen über die Jahre geändert haben könnte. Wir haben den beiden Punkten durch die Verwendung etablierter Routinen zur Analyse von GKV-Daten entgegengewirkt, bspw. indem nur Diagnosen mit 2‑maliger Nennung innerhalb eines Jahres in die Analysen einflossen. Damit ist die Wahrscheinlichkeit hoch, dass trotz der vermuteten erhöhten Sensibilisierung für bspw. Muskel-Skelett-Erkrankungen ein behandlungsbedürftiger Fall als Erkrankungsfall definiert ist, da mindestens 2 Arztbesuche in unterschiedlichen Quartalen kodiert sein mussten. Dieses Vorgehen trägt dazu bei, dass auch Zeittrends robust gemessen werden können.

Die Beschränkung auf das erwerbsfähige Alter kann bei Herz-Kreislauf-Erkrankungen aufgrund der bereits recht niedrigen Inzidenz dazu geführt haben, dass im betrachteten Zeitraum kein Zugewinn an erkrankungsfreien Jahren beobachtet werden konnte. Im Allgemeinen weist die hier betrachtete Gruppe der HKE eine deutlich höhere Fallsterblichkeitsrate (Case Fatality Rate) auf als die der MSE (s. Tabelle S2 im Onlinematerial). Diese Studie fokussiert jedoch weniger auf die Case Fatality, sondern vielmehr auf die Bedeutung der Erkrankung für die Arbeitsfähigkeit und für den vorzeitigen Erwerbsaustritt. Unter diesem Gesichtspunkt sind Analysen zur Entwicklung der erkrankungsfreien Lebenszeit zentral für die Frage nach der weiteren Entwicklung der Gesundheit in der Bevölkerung im erwerbsfähigen Alter.

## Fazit

Die Studie fokussiert auf 2 für die Erwerbsfähigkeit und Frühberentung hochrelevante Erkrankungsgruppen, deren Inzidenz sich in der Bevölkerung über die Zeit konträr entwickelt hat. Während sich die Lebenszeit frei von Muskel-Skelett-Erkrankungen im Zeitverlauf in der Allgemeinbevölkerung und unter den Erwerbstätigen verringert hat, erhöhten sich die Herz-Kreislauf-erkrankungsfreien Jahre leicht. Vor dem Hintergrund der Ausweitung der Lebensarbeitszeit kann das Ergebnis als positiv im Hinblick auf die kardiovaskuläre Gesundheit gedeutet werden. Gleichzeitig zeigen die Ergebnisse, dass wirksame Maßnahmen zur Verbesserung der muskuloskelettalen Gesundheit im erwerbsfähigen Alter dringend benötigt werden.

Die Studie zeigt, wie unterschiedlich sich die erkrankungsfreien Jahre bei MSE und HKE zwischen den Berufsgruppen und Geschlechtern entwickeln. So konnte eine Verringerung der gesundheitlichen Ungleichheit festgestellt werden: Besonders Männer in un- und angelernten Berufen profitieren im Zeitverlauf, was möglicherweise auf verbesserte Arbeitsschutzmaßnahmen und verringerte körperliche Belastung zurückzuführen ist. Unter den Erwerbstätigen in höherqualifizierten Berufen könnten u. a. mehr sitzende Tätigkeiten und eine steigende Arbeitsdichte zu weniger positiven Entwicklungen im Zeitverlauf geführt haben. Diese Einflussfaktoren sollten in weiteren Studien im Detail untersucht werden.

### Supplementary Information




