# Supplementary material for: Healthier at work for longer? Trends in life years free of cardiovascular and musculoskeletal diseases in the employed and general population based on health insurance claims data
Source: Bundesgesundheitsblatt Gesundheitsforschung Gesundheitsschutz. 2024 Apr 12;67(5):555–63. [Article in German] doi: 10.1007/s00103-024-03868-8 (PMC11093846; doi:10.1007/s00103-024-03868-8)
Supplement: Supplementary file 1 [file 103_2024_3868_MOESM1_ESM.pdf]

Bundesgesundheitsblatt

Onlinematerial

Länger gesund im Beruf? Trends in den Lebensjahren frei von Herz-Kreislauf- und Muskel-Skelett-Erkrankungen in GKV-Daten für die Erwerbstätigen- und Allgemeinbevölkerung

Juliane Tetzlaff[1*], Jelena Epping[1]
1 - Medizinische Soziologie, Medizinische Hochschule Hannover, Hannover, Niedersachsen, Deutschland

***Korrespondierende Autorin**
Medizinische Soziologie, Medizinische Hochschule Hannover, Carl-Neuberg Str. 1, 30625 Hannover, Deutschland
E-mail: Tetzlaff.Juliane@mh-hannover.de

Tabelle S1. Anteil der sozialversicherungspflichtig Beschäftigten an der Allgemeinbevölkerung zum 31. Dezember eines Jahres nach Geschlecht und Altersgruppen für die Bundesrepublik und die Versichertenpopulation der AOK Niedersachsen.

		BRD							AOKN						
		2008	2011	2012	2013	2016	2017	2018	2008	2011	2012	2013	2016	2017	2018
männlich	35- unter 45	65%	69%	69%	70%	73%	74%	75%	71%	75%	75%	75%	75%	76%	78%
	45- unter 55	61%	65%	65%	66%	69%	70%	71%	67%	71%	71%	72%	74%	75%	76%
	55- unter 65	41%	46%	47%	49%	52%	54%	56%	48%	52%	53%	54%	58%	60%	62%
weiblich	35- unter 45	54%	58%	59%	59%	63%	64%	65%	47%	51%	52%	53%	58%	59%	62%
	45- unter 55	55%	59%	60%	60%	64%	65%	65%	51%	54%	55%	56%	61%	63%	65%
	55- unter 65	33%	37%	39%	41%	47%	49%	51%	31%	35%	37%	39%	45%	48%	50%

Notiz: Datenquelle Statistisches Bundesamt. Eigene Berechnung der Anteile, eigene Darstellung. Die Daten sind nicht vor 2008 verfügbar. Die Erwerbstätigenzahlen sind ausschließlich in diesen Altersgruppen verfügbar. Die Daten für Niedersachsen sind nicht stratifiziert nach Geschlecht verfügbar.

Tabelle S2. Anzahl der Ereignisse und Übergangsrate (pro 100.000 Personenjahre) für die Allgemeinbevölkerung nach Altersgruppe, Geschlecht und Erkrankung.

		MSE				HKE			
		Männer		Frauen		Männer		Frauen	
Übergang		Anzahl der Ereignisse	Übergangs rate	Anzahl der Ereignisse	Übergangs rate	Anzahl der Ereignisse	Übergangs rate	Anzahl der Ereignisse	Übergangs rate
Inzidenz	30-34	56.025	10.576	54.778	11.514	1.051	165	685	115
	35-39	61.990	12.127	62.597	13.850	2.168	337	1.371	226
	40-44	74.928	13.649	75.185	15.994	4.555	630	2.659	389
	45-49	85.121	14.660	86.431	17.844	9.040	1.135	4.989	650
	50-54	83.707	15.889	84.000	19.587	14.585	1.938	7.854	1.045
	55-59	70.280	16.802	68.918	20.048	18.691	3.007	10.589	1.595
	60-65	59.523	16.565	59.898	19.143	23.141	4.232	15.785	2.453
Genesung (MSE)	30-34	44.060	41.020	43.285	36.681				
	35-39	48.845	35.912	47.771	30.482				
	40-44	58.297	31.207	56.186	25.477				
	45-49	66.171	26.447	64.221	21.508				
	50-54	63.360	21.730	62.351	17.668				
	55-59	52.625	17.307	52.026	14.105				
	60-65	47.149	13.727	46.991	11.259				
Versterben nach Erkrankung	30-34	122	114	59	50	35	2.427	26	2.625
	35-39	167	123	115	73	70	2.422	49	2.540
	40-44	409	219	292	132	148	2.383	84	2.228
	45-49	940	376	563	189	369	2.908	194	2.733
	50-54	1.745	598	1.043	296	747	3.558	357	3.114
	55-59	3.053	1.004	1.778	482	1.163	4.137	531	3.335
	60-65	5.361	1.561	3.194	765	1.907	5.352	942	3.886
Versterben erkrankungs-frei	30-34	441	83	198	42	513	81	223	38
	35-39	740	145	320	71	811	127	367	61
	40-44	1.212	221	571	121	1.380	193	724	106
	45-49	2.271	391	1.005	207	2.550	326	1.273	167
	50-54	3.580	680	1.517	354	3.853	527	1.968	266
	55-59	4.613	1.103	1.955	569	4.856	815	2.676	412
	60-65	6.881	1.915	2.814	899	6.719	1.303	3.833	616

Notiz: Eigene Berechnungen, Datenquelle AOK Niedersachsen. Datenquelle AOK Niedersachsen. Die im Nenner einbezogenen Personenjahre zur Berechnung der Übergangsrate Genesung (MSE) (Jahre nach Inzidenz bis Genesung) unterscheiden sich von den im Nenner einbezogenen Personenjahren Inzidenz (MSE) (erkrankungsfreie Jahre), dies trägt zu Unterschieden in der Höhe der beiden Raten bei.
